# (*E*,*E*)-*N* ^1^-(2,3,4,5,6-Penta­fluoro­benzyl­idene)-*N* ^4^-(3,4,5-trimeth­oxy­benzyl­idene)benzene-1,4-diamine

**DOI:** 10.1107/S160053681104904X

**Published:** 2011-11-23

**Authors:** Alain Collas, Frank Blockhuys

**Affiliations:** a Department of Chemistry, University of Antwerp, Universiteitsplein 1, B-2610 Antwerp, Belgium

## Abstract

The title compound, C_23_H_17_F_5_N_2_O_3_, forms a layered centrosymmetric crystal structure in which C—H⋯F inter­actions are responsible for the formation of planar ribbons along [110], meth­oxy–meth­oxy (C—H⋯O) inter­actions for the formation of layers parallel to [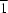
13], and OCH_3_⋯π and C—F⋯π inter­actions for the stacking of these layers.

## Related literature

For asymmetrically substituted *A*–π–*D* distyryl­benzene deriv­atives, see: Bartholomew *et al.* (2000 ▶). For compounds with π-conjugated systems and fluorinated rings, see: Coates *et al.* (1998 ▶); Adamson *et al.* (1994 ▶); Li *et al.* (1994 ▶); Ponzini *et al.* (2000 ▶); Allaway *et al.* (2002 ▶); Collings *et al.* (2004 ▶); Papagni *et al.* (2010 ▶). For structures of related benzyl­idine aniline oligomers, see: Collas, De Borger *et al.* (2011 ▶); Collas, Zeller & Blockhuys (2011 ▶). For a description of the Cambridge Structural Database, see: Allen (2002 ▶).
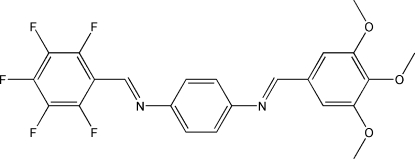


## Experimental

### 

#### Crystal data

C_23_H_17_F_5_N_2_O_3_
*M* *_r_* = 464.39
Triclinic, 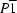

*a* = 7.131 (2) Å
*b* = 11.749 (3) Å
*c* = 12.654 (3) Å
α = 83.85 (2)°
β = 83.12 (2)°
γ = 89.50 (2)°
*V* = 1046.5 (5) Å^3^
*Z* = 2
Mo *K*α radiation
μ = 0.13 mm^−1^
*T* = 298 K
0.3 × 0.2 × 0.2 mm

#### Data collection

Enraf–Nonius CAD-4 diffractometer
7650 measured reflections
3831 independent reflections
2077 reflections with *I* > 2σ(*I*)
*R*_int_ = 0.012
3 standard reflections every 60 min  intensity decay: none

#### Refinement

*R*[*F* ^2^ > 2σ(*F* ^2^)] = 0.078
*wR*(*F* ^2^) = 0.240
*S* = 1.06
3831 reflections
301 parameters
H-atom parameters constrained
Δρ_max_ = 0.25 e Å^−3^
Δρ_min_ = −0.32 e Å^−3^

### 

Data collection: *CAD-4 EXPRESS* (Enraf–Nonius, 1994 ▶); cell refinement: *CAD-4 EXPRESS*; data reduction: *DREAR* (Blessing, 1987 ▶); program(s) used to solve structure: *SHELXS97* (Sheldrick, 2008 ▶); program(s) used to refine structure: *SHELXL97* (Sheldrick, 2008 ▶); molecular graphics: *ORTEP-3* (Farrugia, 1997 ▶) and *Mercury* (Macrae *et al.*, 2008 ▶); software used to prepare material for publication: *WinGX* (Farrugia, 1999 ▶) and *PLATON* (Spek, 2009 ▶).

## Supplementary Material

Crystal structure: contains datablock(s) I, global. DOI: 10.1107/S160053681104904X/zl2429sup1.cif

Structure factors: contains datablock(s) I. DOI: 10.1107/S160053681104904X/zl2429Isup2.hkl

Supplementary material file. DOI: 10.1107/S160053681104904X/zl2429Isup3.cml

Additional supplementary materials:  crystallographic information; 3D view; checkCIF report

## Figures and Tables

**Table 1:** List of relevant short contacts in the crystal packing of the title  compound (Å, °) *Cg*2 and *Cg*3 are the centroids of the C31–C36 and C51–C56 rings, respectively.

*D*—*X*⋯*A*	*D*—*X*	*X*⋯*A*	*D*⋯*A*	*D*—*X*⋯*A*
C33—H33⋯F6^i^	0.93	2.66	3.452 (4)	144
C35—H35⋯F2^ii^	0.93	2.51	3.293 (4)	142
C52—H52⋯F5^i^	0.93	2.49	3.370 (4)	158
C531—H53*B*⋯O53^iii^	0.96	2.63	3.353 (5)	132
C551—H55*B*⋯O55^iv^	0.96	2.72	3.567 (4)	148
C551—H55*C*⋯*Cg*3^v^	0.96	2.78	3.604 (4)	145
C6—F6⋯*Cg*2^vi^	1.33 (1)	3.28 (1)	3.699 (4)	98 (1)
C6—F6⋯*Cg*3^vii^	1.33 (1)	3.34 (1)	3.507 (4)	86 (1)
C541—H54*C*⋯F3^viii^	0.96	2.52	3.430 (5)	158

Symmetry codes: (i) 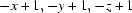; (ii) 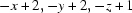; (iii) 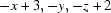; (iv) 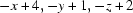; (v) 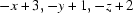; (vi) 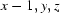; (vii) 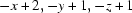; (viii) 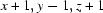.

**Fig. 1.**
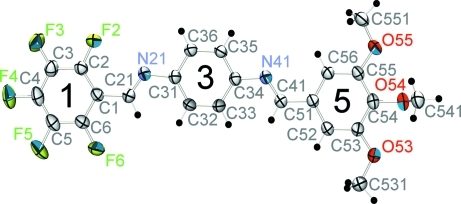
: Molecular structure of the title compound showing the numbering scheme. Displacement ellipsoids are drawn at the 50% probability level; hydrogen atoms are represented by spheres with an arbitrary radius.

**Fig. 2.**
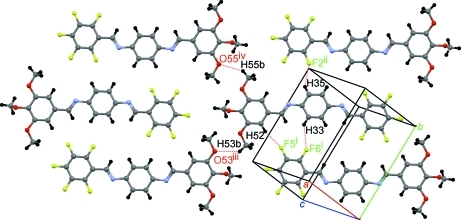
: The CH···F interactions responsible for the formation of the planar ribbons and the CH···O interactions responsible for the formation of layers. See Table 1 for details.

**Fig. 3.**
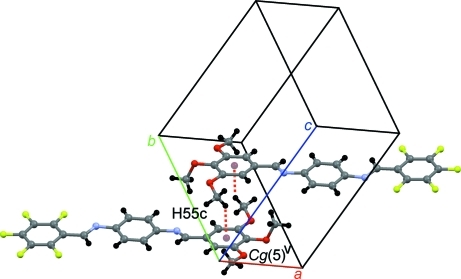
: The CH···π interactions responsible for the stacking of the layers. See Table 1 for details.

**Fig. 4.**
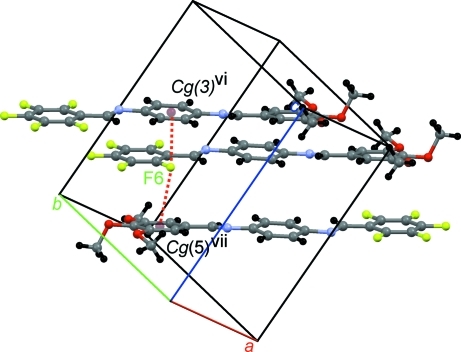
: The CF···π interactions responsible for the stacking of the layers. See Table 1 for details.

## supplementary crystallographic information

### Comment

Even though a perfluorinated aromatic ring is an excellent acceptor moiety for inclusion in asymmetrically substituted push-pull distyrylbenzene derivatives of the A-π-D type, only one such compound (CSD refcode REFKUI), with a *tert*-butyl group as a rather poor electron donor (D) (Bartholomew *et al.*, 2000), is found in the CSD (Allen, 2002). Eight other A-π-D structures containing perfluorinated rings are known, but all of these have π-systems limited to two peripheral rings connected by a spacer: JALLAK, JALKUD and JALKOX have a –C≡C– spacer (Collings *et al.*, 2004), SERQEL (Coates *et al.*, 1998) and NUZVAG (Papagni *et al.*, 2010) are stilbenes, and YOVWUB (Li *et al.*, 1994), WERXEW (Adamson *et al.*, 1994), HUTXUP and BANGOM (Allaway *et al.*, 2002) are benzylidene anilines. Also, the structure of one octupolar star-shaped compound with a benzene ring as the central moiety and –C≡C– spacers (WEVYOL) has been determined (Ponzini *et al.*, 2000). Here, we present the first solid-state structure of an asymmetrically substituted push-pull benzylidene aniline derivative with a more extended conjugated system.

The title compound, (I), can be easily obtained from the condensation of 3,4,5-trimethoxybenzaldehyde with *E*-*N*-pentafluorobenzylidene-1,4-phenylenediamine, (II), which can be prepared from pentafluorobenzaldehyde and 1,4-phenylenediamine, but only at lower temperatures, considering that the extreme activation of the carbonyl group in the latter benzaldehyde would otherwise lead to the symmetrical bis(benzylidene aniline). (I) crystallizes as quasi-planar molecules in the centrosymmetric space group *P*1: the dihedral angle between the l.s. planes of rings 1 and 3 (Fig. 1) is 2.58 (17)°, while the one between rings 3 and 5 is 7.81 (17)°. Molecules of (I) are found in the *syn* conformation in which both imine spacers point in the same direction.

The crystal packing features relatively flat ribbons of oligomers held together by three CH···F interactions (Fig. 2 and Table 1, entries 1–3). These ribbons are then fused by two mutual methoxy···methoxy (CH···O) interactions to form layers (Fig. 2 and Table 1, entries 4 and 5). Finally, the layers of quasi-planar molecules are stacked by dint of three interactions involving the π-systems of the electron-rich aromatic rings, *i.e.*, the central and the methoxy-substituted rings [with centroids *Cg*(3) and *Cg*(5), respectively]. First, a mutual OCH_3_···π interaction initiated by the methoxy groups in the 5-position exists between the layers (Fig. 3 and Table 1, entry 6). Then, the weakly polarizable fluorine atom F6 simultaneously contacts both above mentioned centroids (Fig. 4 and Table 1, entries 7 and 8). Finally, the methoxy group in the 4-position (located out of the plane of the rest of the molecule) participates in a CH···F weak hydrogen bond (Table 1, entry 9; not given in the Figures). Thus, all methoxy groups and all fluorine atoms except F4 are used in the supramolecular arrangement.

The nitrogen atoms in the imine spacers and the activated azomethine hydrogen atoms H21 and H41 do not participate in any intermolecular contacts. This may be linked to the *absence* of the typical twist of the central phenylenediamine ring of about 40° out of the planes of the spacers and the peripheral rings [for recent examples, see: Collas, De Borger, Amanova & Blockhuys (2011) and Collas, Zeller & Blockhuys (2011)], as the availability of the nitrogen atom in (I) is reduced. On the other hand, the resulting planar conformation leads to an improved stacking of molecules, notwithstanding the modest packing efficiency of only 68.3%.

### Experimental

All reagents and solvents were obtained from ACROS and used as received, except for pentafluorobenzaldehyde which was purchased from Fluorochem Ltd. All NMR spectra were recorded in CDCl_3_ on a Bruker Avance II spectrometer at frequencies of 400 MHz for ^1^H and 100 MHz for ^13^C with tetramethylsilane (TMS) as internal standard. Chemical shifts are given in p.p.m. and coupling constants J in Hz. Melting points were obtained with an open capillary electrothermal melting point apparatus and are uncorrected.

*E*-*N*-Pentafluorobenzylidene-1,4-phenylenediamine (II). To a cooled solution (233 K) of 1,4-phenylenediamine (2.2 g, 0.02 mol) in CH_2_Cl_2_ (100 ml) was added dropwise a solution of pentafluorobenzaldehyde (4.0 g, 0.02 mol). After addition, the mixture was allowed to warm up to room temperature. The yellow precipitate was collected *via* filtration and washed with diethyl ether. The yield was 4.3 g (0.015 mol, 73%). *M*.p. 460 K. δ^1^H 3.80 (s, 2H, NH_2_), 6.69 (d, ^3^J = 8.6, 2H, H33 and H35), 7.18 (d, ^3^J = 8.6, 2H, H32 and H36), 8.57 (s, 1H, H21). δ^13^C 112.06 (m, C1), 115.41 (C33 and C35), 122.79 (C32 and C36), 137.82 (m, ^1^J_CF_ = 253, C3 and C5), 141.93 (m, ^1^J_CF_ = 258, C4), 142.03 (C31), 146.00 (m, ^1^J_CF_ = 261, C2 and C6), 146.72 (C34), 150.37 (C21).

(*E*,*E*)-*N*-(3,4,5-Trimethoxybenzylidene)-*N*'- (pentafluorobenzylidene)-1,4-phenylenediamine (I). To a solution of (II) (285 mg, 1 mmol) in methanol was added 3,4,5-trimethoxybenzaldehyde (200 mg, 1 mmol). The solution was left to stir overnight and the resulting yellow powder was collected *via* filtration and washed with cold methanol. The yield was 299 mg (0.64 mmol, 64%). *M*.p. 482 K. δ^1^H 3.92 (s, 3H, 4-OCH_3_), 3.95 (s, 6H, 3-OCH_3_ and 5-OCH_3_), 7.17 (s, 2H, H52 and H56), 7.27 (s, 2H, H33 and H35), 7.30 (s, 2H, H32 and H36), 8.40 (s, 1H, H41), 8.60 (s, 1H, H21). δ^13^C 56.30 (4-OCH_3_), 60.99 (3-OCH_3_ and 5-OCH_3_), 105.89 (C52 and C56), 111.31 (C1), 121.84 (C33 and C35), 122.02 (C32 and C36), 131.81 (C51), 141.13 (C54), 149.86 (C34), 150.35 (C31), 153.59 (C53 and C55), 159.17 (C41). Due to the low solubility of (I) and the signal broadening associated with the (long-range) coupling with the ^19^F nuclei, signals for C2, C3, C4, C5, C6 and C21 can not be distinguished from the noise in the ^13^C spectrum. Suitable crystals were grown by the slow evaporation of an acetone solution.

### Crystal data

**Table:**

C_23_H_17_F_5_N_2_O_3_	*Z* = 2
*M**_r_* = 464.39	*F*(000) = 476
Triclinic, *P*1	*D*_x_ = 1.474 Mg m^−^^3^
Hall symbol: -P 1	Mo *K*α radiation, λ = 0.71073 Å
*a* = 7.131 (2) Å	Cell parameters from 25 reflections
*b* = 11.749 (3) Å	θ = 5.8–20.8°
*c* = 12.654 (3) Å	µ = 0.13 mm^−^^1^
α = 83.85 (2)°	*T* = 298 K
β = 83.12 (2)°	Prism, orange
γ = 89.50 (2)°	0.3 × 0.2 × 0.2 mm
*V* = 1046.5 (5) Å^3^	

### Data collection

**Table:**

Enraf–Nonius CAD-4 diffractometer	*R*_int_ = 0.012
Radiation source: fine-focus sealed tube	θ_max_ = 25.3°, θ_min_ = 1.6°
graphite	*h* = −8→8
θ/2ω scans	*k* = −14→14
7650 measured reflections	*l* = −15→15
3831 independent reflections	3 standard reflections every 60 min
2077 reflections with *I* > 2σ(*I*)	intensity decay: none

### Refinement

**Table:**

Refinement on *F*^2^	Primary atom site location: structure-invariant direct methods
Least-squares matrix: full	Secondary atom site location: difference Fourier map
*R*[*F*^2^ > 2σ(*F*^2^)] = 0.078	Hydrogen site location: inferred from neighbouring sites
*wR*(*F*^2^) = 0.240	H-atom parameters constrained
*S* = 1.06	*w* = 1/[σ^2^(*F*_o_^2^) + (0.1397*P*)^2^ + 0.0922*P*] where *P* = (*F*_o_^2^ + 2*F*_c_^2^)/3
3831 reflections	(Δ/σ)_max_ < 0.001
301 parameters	Δρ_max_ = 0.25 e Å^−^^3^
0 restraints	Δρ_min_ = −0.32 e Å^−^^3^

### Special details

**Table:**

Geometry. All e.s.d.'s (except the e.s.d. in the dihedral angle between two l.s. planes) are estimated using the full covariance matrix. The cell e.s.d.'s are taken into account individually in the estimation of e.s.d.'s in distances, angles and torsion angles; correlations between e.s.d.'s in cell parameters are only used when they are defined by crystal symmetry. An approximate (isotropic) treatment of cell e.s.d.'s is used for estimating e.s.d.'s involving l.s. planes.
Refinement. Refinement of *F*^2^ against ALL reflections. The weighted *R*-factor *wR* and goodness of fit *S* are based on *F*^2^, conventional *R*-factors *R* are based on *F*, with *F* set to zero for negative *F*^2^. The threshold expression of *F*^2^ > σ(*F*^2^) is used only for calculating *R*-factors(gt) *etc*. and is not relevant to the choice of reflections for refinement. *R*-factors based on *F*^2^ are statistically about twice as large as those based on *F*, and *R*-factors based on ALL data will be even larger. Reflection -1 1 3 was omitted.

### Fractional atomic coordinates and isotropic or equivalent isotropic displacement parameters (Å^2^)

**Table:**

	*x*	*y*	*z*	*U*_iso_*/*U*_eq_	
C1	0.4594 (4)	0.8471 (3)	0.3807 (2)	0.0644 (8)	
C2	0.4713 (5)	0.9623 (3)	0.3488 (3)	0.0699 (9)	
C3	0.3407 (6)	1.0190 (3)	0.2900 (3)	0.0826 (10)	
C4	0.1929 (5)	0.9579 (4)	0.2629 (3)	0.0885 (12)	
C5	0.1764 (5)	0.8446 (4)	0.2928 (3)	0.0822 (10)	
C6	0.3086 (5)	0.7896 (3)	0.3501 (3)	0.0706 (9)	
C31	0.8512 (5)	0.7527 (3)	0.5358 (3)	0.0736 (9)	
C32	0.8325 (6)	0.6361 (3)	0.5613 (4)	0.1033 (15)	
H32	0.7300	0.5986	0.5415	0.124*	
C33	0.9613 (6)	0.5748 (3)	0.6149 (3)	0.0974 (14)	
H33	0.9463	0.4960	0.6304	0.117*	
C34	1.1146 (4)	0.6285 (3)	0.6469 (2)	0.0632 (8)	
C35	1.1320 (4)	0.7443 (3)	0.6226 (2)	0.0675 (8)	
H35	1.2336	0.7818	0.6435	0.081*	
C36	1.0026 (4)	0.8074 (3)	0.5677 (3)	0.0692 (9)	
H36	1.0173	0.8862	0.5523	0.083*	
F2	0.6128 (3)	1.02449 (18)	0.3725 (2)	0.1006 (8)	
F3	0.3585 (4)	1.1305 (2)	0.2600 (2)	0.1206 (9)	
F4	0.0656 (4)	1.0128 (3)	0.2066 (2)	0.1289 (10)	
F5	0.0318 (3)	0.7845 (3)	0.26703 (19)	0.1193 (9)	
F6	0.2870 (3)	0.67739 (19)	0.37783 (18)	0.0951 (7)	
C11	0.5942 (5)	0.7820 (3)	0.4420 (3)	0.0742 (9)	
H11	0.5780	0.7030	0.4540	0.089*	
C41	1.2322 (5)	0.4731 (3)	0.7473 (3)	0.0667 (8)	
H41	1.1210	0.4351	0.7408	0.080*	
C51	1.3720 (4)	0.4127 (3)	0.8089 (2)	0.0631 (8)	
C52	1.3406 (5)	0.2992 (3)	0.8467 (3)	0.0691 (9)	
H52	1.2324	0.2625	0.8330	0.083*	
C53	1.4695 (5)	0.2393 (3)	0.9049 (3)	0.0684 (8)	
C54	1.6325 (4)	0.2948 (3)	0.9237 (3)	0.0691 (9)	
C55	1.6611 (4)	0.4107 (3)	0.8878 (3)	0.0660 (8)	
C56	1.5323 (4)	0.4700 (3)	0.8289 (2)	0.0666 (8)	
H56	1.5525	0.5466	0.8033	0.080*	
C531	1.2776 (6)	0.0728 (3)	0.9403 (4)	0.1006 (13)	
H53A	1.1758	0.1170	0.9717	0.151*	
H53B	1.2771	−0.0021	0.9790	0.151*	
H53C	1.2618	0.0662	0.8670	0.151*	
C541	1.7388 (6)	0.2398 (4)	1.0885 (3)	0.0918 (11)	
H54A	1.7360	0.3182	1.1034	0.138*	
H54B	1.8407	0.2010	1.1203	0.138*	
H54C	1.6214	0.2033	1.1179	0.138*	
C551	1.8259 (5)	0.5788 (4)	0.9087 (3)	0.0869 (11)	
H55A	1.8477	0.6104	0.8351	0.130*	
H55B	1.9264	0.6015	0.9461	0.130*	
H55C	1.7079	0.6066	0.9409	0.130*	
N11	0.7261 (4)	0.8232 (3)	0.4786 (2)	0.0855 (9)	
N41	1.2556 (4)	0.5735 (2)	0.7032 (2)	0.0688 (7)	
O53	1.4518 (4)	0.1280 (2)	0.9453 (2)	0.0898 (8)	
O54	1.7660 (3)	0.2351 (2)	0.97682 (19)	0.0817 (7)	
O55	1.8193 (3)	0.4583 (2)	0.9145 (2)	0.0844 (7)	

### Atomic displacement parameters (Å^2^)

**Table:**

	*U*^11^	*U*^22^	*U*^33^	*U*^12^	*U*^13^	*U*^23^
C1	0.0601 (18)	0.075 (2)	0.0590 (17)	−0.0068 (16)	−0.0117 (14)	−0.0037 (15)
C2	0.0618 (19)	0.074 (2)	0.072 (2)	−0.0107 (16)	−0.0097 (16)	0.0037 (16)
C3	0.082 (2)	0.088 (3)	0.072 (2)	0.004 (2)	−0.0062 (19)	0.0133 (19)
C4	0.070 (2)	0.132 (4)	0.062 (2)	0.012 (2)	−0.0185 (17)	0.002 (2)
C5	0.067 (2)	0.118 (3)	0.066 (2)	−0.005 (2)	−0.0178 (17)	−0.016 (2)
C6	0.067 (2)	0.081 (2)	0.0649 (19)	−0.0119 (17)	−0.0087 (16)	−0.0101 (16)
C31	0.078 (2)	0.071 (2)	0.074 (2)	−0.0098 (17)	−0.0294 (17)	0.0077 (16)
C32	0.108 (3)	0.072 (2)	0.139 (3)	−0.032 (2)	−0.076 (3)	0.018 (2)
C33	0.115 (3)	0.064 (2)	0.121 (3)	−0.022 (2)	−0.069 (3)	0.018 (2)
C34	0.0668 (19)	0.0662 (19)	0.0578 (17)	−0.0078 (15)	−0.0177 (14)	−0.0004 (14)
C35	0.0650 (19)	0.071 (2)	0.0672 (18)	−0.0177 (16)	−0.0186 (15)	0.0032 (15)
C36	0.072 (2)	0.0628 (18)	0.072 (2)	−0.0137 (16)	−0.0174 (16)	0.0068 (15)
F2	0.0859 (14)	0.0770 (13)	0.1376 (19)	−0.0243 (11)	−0.0311 (13)	0.0158 (12)
F3	0.121 (2)	0.0973 (17)	0.131 (2)	0.0054 (14)	−0.0103 (16)	0.0430 (15)
F4	0.1015 (18)	0.188 (3)	0.0967 (16)	0.0282 (17)	−0.0426 (14)	0.0170 (16)
F5	0.0858 (15)	0.172 (3)	0.1109 (17)	−0.0216 (16)	−0.0427 (13)	−0.0292 (16)
F6	0.0956 (15)	0.0853 (15)	0.1084 (16)	−0.0248 (12)	−0.0244 (12)	−0.0126 (12)
C11	0.075 (2)	0.067 (2)	0.082 (2)	−0.0064 (17)	−0.0232 (18)	0.0049 (16)
C41	0.0615 (19)	0.067 (2)	0.073 (2)	−0.0038 (16)	−0.0170 (15)	−0.0019 (16)
C51	0.0565 (18)	0.070 (2)	0.0637 (18)	0.0001 (15)	−0.0139 (14)	−0.0060 (15)
C52	0.068 (2)	0.070 (2)	0.074 (2)	−0.0008 (16)	−0.0261 (16)	−0.0082 (16)
C53	0.073 (2)	0.0602 (19)	0.076 (2)	0.0048 (16)	−0.0223 (17)	−0.0072 (16)
C54	0.0604 (19)	0.079 (2)	0.0696 (19)	0.0111 (17)	−0.0157 (15)	−0.0082 (16)
C55	0.0497 (17)	0.080 (2)	0.0693 (19)	−0.0018 (15)	−0.0116 (14)	−0.0077 (16)
C56	0.0579 (19)	0.071 (2)	0.070 (2)	−0.0019 (15)	−0.0103 (15)	−0.0008 (15)
C531	0.105 (3)	0.074 (2)	0.124 (3)	−0.016 (2)	−0.041 (3)	0.013 (2)
C541	0.092 (3)	0.104 (3)	0.082 (3)	−0.005 (2)	−0.034 (2)	0.004 (2)
C551	0.068 (2)	0.101 (3)	0.093 (3)	−0.019 (2)	−0.0210 (19)	−0.008 (2)
N11	0.086 (2)	0.0748 (18)	0.100 (2)	−0.0144 (16)	−0.0445 (18)	0.0129 (15)
N41	0.0686 (16)	0.0697 (17)	0.0693 (16)	−0.0082 (13)	−0.0220 (13)	0.0030 (13)
O53	0.0953 (18)	0.0672 (15)	0.112 (2)	−0.0011 (13)	−0.0462 (15)	0.0038 (13)
O54	0.0683 (15)	0.0948 (17)	0.0846 (16)	0.0174 (12)	−0.0264 (12)	−0.0040 (13)
O55	0.0548 (13)	0.0951 (18)	0.1049 (18)	−0.0099 (12)	−0.0239 (12)	−0.0016 (14)

### Geometric parameters (Å, °)

**Table:**

C1—C2	1.371 (4)	C41—N41	1.253 (4)
C1—C6	1.390 (4)	C41—C51	1.470 (4)
C1—C11	1.466 (4)	C41—H41	0.9300
C2—F2	1.332 (4)	C51—C52	1.378 (4)
C2—C3	1.383 (5)	C51—C56	1.394 (4)
C3—F3	1.327 (4)	C52—C53	1.386 (4)
C3—C4	1.377 (6)	C52—H52	0.9300
C4—F4	1.339 (4)	C53—O53	1.353 (4)
C4—C5	1.347 (6)	C53—C54	1.395 (5)
C5—F5	1.346 (4)	C54—O54	1.375 (4)
C5—C6	1.372 (5)	C54—C55	1.397 (5)
C6—F6	1.333 (4)	C55—O55	1.358 (4)
C31—C32	1.376 (5)	C55—C56	1.388 (4)
C31—C36	1.384 (4)	C56—H56	0.9300
C31—N11	1.421 (4)	C531—O53	1.418 (4)
C32—C33	1.363 (5)	C531—H53A	0.9600
C32—H32	0.9300	C531—H53B	0.9600
C33—C34	1.389 (5)	C531—H53C	0.9600
C33—H33	0.9300	C541—O54	1.410 (4)
C34—C35	1.366 (4)	C541—H54A	0.9600
C34—N41	1.414 (4)	C541—H54B	0.9600
C35—C36	1.383 (4)	C541—H54C	0.9600
C35—H35	0.9300	C551—O55	1.411 (5)
C36—H36	0.9300	C551—H55A	0.9600
C11—N11	1.223 (4)	C551—H55B	0.9600
C11—H11	0.9300	C551—H55C	0.9600
			
C2—C1—C6	116.2 (3)	C52—C51—C56	121.0 (3)
C2—C1—C11	124.9 (3)	C52—C51—C41	118.8 (3)
C6—C1—C11	118.9 (3)	C56—C51—C41	120.2 (3)
F2—C2—C1	120.7 (3)	C51—C52—C53	120.4 (3)
F2—C2—C3	117.0 (3)	C51—C52—H52	119.8
C1—C2—C3	122.3 (3)	C53—C52—H52	119.8
F3—C3—C4	120.7 (3)	O53—C53—C52	124.9 (3)
F3—C3—C2	120.3 (4)	O53—C53—C54	115.8 (3)
C4—C3—C2	119.0 (4)	C52—C53—C54	119.3 (3)
F4—C4—C5	120.5 (4)	O54—C54—C53	120.0 (3)
F4—C4—C3	119.1 (4)	O54—C54—C55	120.0 (3)
C5—C4—C3	120.4 (3)	C53—C54—C55	120.1 (3)
F5—C5—C4	120.7 (3)	O55—C55—C56	124.0 (3)
F5—C5—C6	119.5 (4)	O55—C55—C54	115.6 (3)
C4—C5—C6	119.7 (3)	C56—C55—C54	120.4 (3)
F6—C6—C5	117.7 (3)	C55—C56—C51	118.8 (3)
F6—C6—C1	119.9 (3)	C55—C56—H56	120.6
C5—C6—C1	122.4 (3)	C51—C56—H56	120.6
C32—C31—C36	118.5 (3)	O53—C531—H53A	109.5
C32—C31—N11	125.2 (3)	O53—C531—H53B	109.5
C36—C31—N11	116.3 (3)	H53A—C531—H53B	109.5
C33—C32—C31	121.3 (3)	O53—C531—H53C	109.5
C33—C32—H32	119.4	H53A—C531—H53C	109.5
C31—C32—H32	119.4	H53B—C531—H53C	109.5
C32—C33—C34	120.8 (3)	O54—C541—H54A	109.5
C32—C33—H33	119.6	O54—C541—H54B	109.5
C34—C33—H33	119.6	H54A—C541—H54B	109.5
C35—C34—C33	117.9 (3)	O54—C541—H54C	109.5
C35—C34—N41	116.6 (3)	H54A—C541—H54C	109.5
C33—C34—N41	125.5 (3)	H54B—C541—H54C	109.5
C34—C35—C36	121.8 (3)	O55—C551—H55A	109.5
C34—C35—H35	119.1	O55—C551—H55B	109.5
C36—C35—H35	119.1	H55A—C551—H55B	109.5
C35—C36—C31	119.7 (3)	O55—C551—H55C	109.5
C35—C36—H36	120.1	H55A—C551—H55C	109.5
C31—C36—H36	120.1	H55B—C551—H55C	109.5
N11—C11—C1	125.2 (3)	C11—N11—C31	121.1 (3)
N11—C11—H11	117.4	C41—N41—C34	120.8 (3)
C1—C11—H11	117.4	C53—O53—C531	117.5 (3)
N41—C41—C51	123.2 (3)	C54—O54—C541	113.5 (3)
N41—C41—H41	118.4	C55—O55—C551	117.1 (3)
C51—C41—H41	118.4		
			
C6—C1—C2—F2	−178.8 (3)	N11—C31—C36—C35	−179.2 (3)
C11—C1—C2—F2	0.1 (5)	C2—C1—C11—N11	5.4 (6)
C6—C1—C2—C3	0.4 (5)	C6—C1—C11—N11	−175.7 (3)
C11—C1—C2—C3	179.3 (3)	N41—C41—C51—C52	−174.5 (3)
F2—C2—C3—F3	−0.3 (5)	N41—C41—C51—C56	5.9 (5)
C1—C2—C3—F3	−179.5 (3)	C56—C51—C52—C53	−0.5 (5)
F2—C2—C3—C4	179.5 (3)	C41—C51—C52—C53	179.9 (3)
C1—C2—C3—C4	0.3 (6)	C51—C52—C53—O53	−180.0 (3)
F3—C3—C4—F4	−0.6 (6)	C51—C52—C53—C54	−0.8 (5)
C2—C3—C4—F4	179.6 (3)	O53—C53—C54—O54	2.4 (5)
F3—C3—C4—C5	179.5 (3)	C52—C53—C54—O54	−176.9 (3)
C2—C3—C4—C5	−0.3 (6)	O53—C53—C54—C55	−178.2 (3)
F4—C4—C5—F5	−0.2 (6)	C52—C53—C54—C55	2.6 (5)
C3—C4—C5—F5	179.7 (3)	O54—C54—C55—O55	−3.7 (4)
F4—C4—C5—C6	179.7 (3)	C53—C54—C55—O55	176.9 (3)
C3—C4—C5—C6	−0.4 (6)	O54—C54—C55—C56	176.4 (3)
F5—C5—C6—F6	0.0 (5)	C53—C54—C55—C56	−3.0 (5)
C4—C5—C6—F6	−179.8 (3)	O55—C55—C56—C51	−178.2 (3)
F5—C5—C6—C1	−178.9 (3)	C54—C55—C56—C51	1.7 (5)
C4—C5—C6—C1	1.2 (5)	C52—C51—C56—C55	0.1 (5)
C2—C1—C6—F6	179.9 (3)	C41—C51—C56—C55	179.7 (3)
C11—C1—C6—F6	0.9 (5)	C1—C11—N11—C31	−179.0 (3)
C2—C1—C6—C5	−1.1 (5)	C32—C31—N11—C11	−4.2 (6)
C11—C1—C6—C5	179.9 (3)	C36—C31—N11—C11	176.0 (3)
C36—C31—C32—C33	−1.3 (7)	C51—C41—N41—C34	−178.6 (3)
N11—C31—C32—C33	178.9 (4)	C35—C34—N41—C41	165.6 (3)
C31—C32—C33—C34	0.9 (8)	C33—C34—N41—C41	−14.3 (6)
C32—C33—C34—C35	−0.2 (6)	C52—C53—O53—C531	−9.0 (5)
C32—C33—C34—N41	179.7 (4)	C54—C53—O53—C531	171.9 (3)
C33—C34—C35—C36	−0.1 (5)	C53—C54—O54—C541	−91.7 (4)
N41—C34—C35—C36	180.0 (3)	C55—C54—O54—C541	88.9 (4)
C34—C35—C36—C31	−0.3 (5)	C56—C55—O55—C551	18.3 (5)
C32—C31—C36—C35	0.9 (6)	C54—C55—O55—C551	−161.6 (3)

### Table 1 List of relevant short contacts in the crystal packing of the title compound (Å, °).

**Table:**

Entry	D	X	A	X···A	D–X···A
1	C33	H33	F6^i^	2.66	144
2	C35	H35	F2^ii^	2.51	142
3	C52	H52	F5^i^	2.49	158
4	C531	H53b	O53^iii^	2.63	132
5	C551	H55b	O55^iv^	2.72	148
6	C551	H55c	Cg(5)^v^	2.78	145
7	C6	F6	Cg(3)^vi^	3.276 (3)	97.71 (19)
8	C6	F6	Cg(5)^vii^	3.335 (3)	86.15 (19)
9	C541	H54c	F3^viii^	2.52	188

Symmetry codes: (i) 1 – x, 1 – y, 1– z; (ii) 2 – x, 2 – y, 1 – z; (iii) 3 – x, – y, 2 – z; (iv) 4 – x, 1 – y, 2 – z; (v) –3 – x, 1 – y, 2 – z; (vi) –1 + x, y, z; (vii) 2 – x, 1 – y, 1 – z; (viii) 1 + x, –1 + y, 1 + z.

## References

[bb1] Adamson, A. J., Archambeau, Y., Banks, R. E., Beagley, B., Helliwell, M., Pritchard, R. G. & Tipping, A. E. (1994). *Acta Cryst.* C**50**, 967–971.

[bb2] Allaway, C. L., Daly, M., Nieuwenhuizen, M. & Saunder, G. C. (2002). *J. Fluorine Chem.* **115**, 91–99.

[bb3] Allen, F. H. (2002). *Acta Cryst.* B**58**, 380–388. 10.1107/s0108768102003890 12037359

[bb4] Bartholomew, G. P., Baza, G. C., Bu, X. H. & Lachicotte, R. J. (2000). *Chem. Mater.* **12**, 1422–1430.

[bb5] Blessing, R. (1987). *Crystallogr. Rev.* **1**, 3–58.

[bb6] Coates, G., Dunn, A., Henling, L., Ziller, J., Lobkovsky, E. & Grubbs, R. (1998). *J. Am. Chem. Soc.* **120**, 3641–3649.

[bb7] Collas, A., De Borger, R., Amanova, T. & Blockhuys, F. (2011). *CrystEngComm*, **13**, 702–710.

[bb8] Collas, A., Zeller, M. & Blockhuys, F. (2011). *Acta Cryst.* C**67**, o171–o174. 10.1107/S0108270111010109 21540543

[bb9] Collings, J. C., Smith, P. S., Yufit, D. S., Batsanov, A. S., Howard, J. A. K. & Marder, T. B. (2004). *CrystEngComm*, **6**, 25–28.

[bb10] Enraf–Nonius (1994). *CAD-4 EXPRESS* Enraf–Nonius, Delft, The Netherlands.

[bb11] Farrugia, L. J. (1997). *J. Appl. Cryst.* **30**, 565.

[bb12] Farrugia, L. J. (1999). *J. Appl. Cryst.* **32**, 837–838.

[bb13] Li, A. W., Bin, X., Zhu, S. Z., Huang, Q. C. & Liu, J. S. (1994). *J. Fluorine Chem.* **68**, 145–148.

[bb14] Macrae, C. F., Bruno, I. J., Chisholm, J. A., Edgington, P. R., McCabe, P., Pidcock, E., Rodriguez-Monge, L., Taylor, R., van de Streek, J. & Wood, P. A. (2008). *J. Appl. Cryst.* **41**, 466–470.

[bb15] Papagni, A., Del Buttero, P., Bertarelli, C., Miozzo, L., Moret, M., Pryce, M. T. & Rizzato, S. (2010). *New J. Chem.* **34**, 2612–2621.

[bb16] Ponzini, F., Zagha, R., Hardcastle, K. & Siegel, J. S. (2000). *Angew. Chem. Int. Ed.* **39**, 2323–2325. 10.1002/1521-3773(20000703)39:13<2323::aid-anie2323>3.0.co;2-x 10941078

[bb17] Sheldrick, G. M. (2008). *Acta Cryst.* A**64**, 112–122. 10.1107/S0108767307043930 18156677

[bb18] Spek, A. L. (2009). *Acta Cryst.* D**65**, 148–155. 10.1107/S090744490804362X PMC2631630 19171970

